# Simulation of the Diffusion Characteristics of Multifunctional Nanocarriers in Tumor Tissues Using Lattice Gas Automata and the Lattice Boltzmann Method

**DOI:** 10.3390/bioengineering12040429

**Published:** 2025-04-18

**Authors:** Yuming Qin, Kai Yue, Xiaoling Yu, Yu You, Chao Yang, Xinxin Zhang

**Affiliations:** 1 School of Energy and Environmental Engineering, University of Science and Technology Beijing, Beijing 100083, China; 2 Shunde Innovation School, University of Science and Technology Beijing, Foshan 528399, China

**Keywords:** diffusion, lattice gas automata, lattice Boltzmann method, tumor tissues, nanoparticles, thermogenesis

## Abstract

Understanding the diffusion mechanisms of nanocarriers in tumor tissues is crucial for enhancing drug delivery to target areas. This study developed a simulation method combining lattice gas automata and the lattice Boltzmann method to explore the diffusion behaviors of ligand-coated nanoparticles (NPs) in the extracellular matrix (ECM) and tumor tissues under the influence of external fields. We propose mathematical models to describe how the movement of NPs is affected by thermomagnetic effects and by their interactions with ECM fiber walls and cells, and to calculate the flow field and temperature distribution in tumor tissues containing interstitial fluids. The results show that reduced tissue porosity and increased ECM fiber and cell densities hinder NP transport. Conversely, degrading ECM collagen fibers with thermal or other energy forms significantly improved NP diffusion in treated tissues. Modifying the surface zeta potential of NPs allowed for the regulation of NP adhesion to ECM fibers and cell membranes based on their charged components. However, altering the charge on the NP surface did not further enhance diffusion once a certain charge level was reached. Increased temperatures from NP heat generation under external fields improved interstitial fluid flow, thereby enhancing NP diffusion. Additionally, a static magnetic field gradient considerably increased the penetration depth of magnetic NPs in the direction of the field, with minimal effects on diffusion in other directions and, in some cases, reducing diffusion.

## 1. Introduction

Nanoparticles (NPs) have gained significant attention due to their high environmental sensitivity, lesion-targeting ability, and drug-delivery capability, which lead to great potential in diagnosis and therapy for diseases [1,2,3]. Recent advances in functionalized nanoparticles, such as those used in MRI-guided therapies, photothermal therapy, and immunotherapy, have demonstrated their transformative potential in oncology [4,5]. For effective treatment, it is necessary for NPs to penetrate deeply into pathological tissues. However, NP penetration is limited by various factors in the lesion environment, such as high-density cell accumulation, high interstitial fluid pressure, abnormal vascular structure, and a dense extracellular matrix (ECM) [6,7], resulting in poor drug utilization and treatment efficacy [8,9]. Notably, recent studies have revealed that NP accumulation may induce DNA damage through reactive oxygen species generation and direct nucleobase interactions [10]. This biosafety concern underscores the urgency to enhance NP diffusion efficiency, which can lower dosage requirements without compromising therapeutic outcomes, ultimately reducing DNA damage risks. To overcome these challenges, it is essential to explore the mechanisms of NP diffusion in lesions in depth, to provide important insights for designing NPs with better therapeutic effects.

The diffusion efficiency of NPs in lesions can be enhanced by several methods, which have been researched in recent experiments. By killing tumor cells [11], normalizing the tumor vasculature and ECM [12], and degrading ECM [13], the permeability of tumor tissue is increased, improving the uniformity of macromolecular diffusion in tumor tissue. In addition, modification of specific surface materials (e.g., surface charge modulation [14] and hydrophobicity adjustment [14,15]) and the application of external fields (e.g., magnetic guidance in MRI-based therapies [4,5] and near-infrared–driven [16,17]) have been proven to increase the depth of penetration of NPs. However, there is some controversy over the effect of NP characteristics on diffusion efficiency. For example, Wang et al. [18] demonstrated in murine models of breast and prostate cancer that NPs with positive surface charges exhibited significantly higher tumor penetration. But Jin et al. [19] employed the MCF-7 human breast cancer cell line to construct multicellular tumor spheroids (MCTS) and demonstrated that NPs with negative surface charges exhibited enhanced tumor penetration and more uniform distribution compared to positively charged nanorods. Several contradictory studies have been published [20,21,22,23]. One possible explanation is that variations in the electrical properties of tumor cell and ECM surfaces lead nanoparticles with similar surface charges to exhibit opposing diffusion behaviors in different tumor microenvironments. The existing research focuses on describing experimental phenomena, but lacks consideration of the interactions between NPs and the environment, as well as understanding the mechanism of diffusion, which leads to difficulties in explaining these contradictions.

Numerical simulation methods have been applied to study the mechanism of NP diffusion in the human body, allowing for in-depth analysis of NP diffusion effects in human tissues. Golneshan et al. [24] constructed a biothermal model and simulated diffusion of magnetic hyperthermia-inducing NPs delivered to tumor tissues via different injection methods, and clarified the impact of injection method on NP diffusion based on numerical simulation data. In addition, studying the motion of individual particles can help to establish a diffusion model for a collection of particles in tissues [25,26,27]. Yuan et al. [28] developed a framework by combining a random anisotropic geometric model and a mathematical particle tracking model to study the movement of NPs in brain white matter. The trajectories of NPs and the flow field distribution were calculated by finite element methods. Complex porous structures with anisotropic characteristics exist in various lesions, like tumor tissues and diseased white matter. To evaluate the permeation range, it is essential to reconstruct tumor tissue as an anisotropic porous structure and analyze the movement of each nanoparticle. However, compared to traditional methods such as the finite element method, the LB method has the advantage of dealing with complex boundary conditions, so that it can couple different physical fields by introducing different collision models or interaction mechanisms [29,30,31]. The LB method can establish a high-precision model of the diffusion process of NPs in disease tissues, which is expected to provide valuable information for studying the diffusion mechanism of NPs.

This study aimed to propose a mesoscopic-scale numerical simulation method to investigate the diffusion mechanism of NPs in tumor tissues. Three-dimensional geometrical models of the ECM and tumor tissues were constructed to simulate the anisotropic, multiphase, and heterogeneous properties of tissues. Furthermore, this study introduces an innovative hybrid computational framework integrating the lattice Boltzmann method (LBM) for multiphase flow modeling and lattice gas automata (LGA) for discrete particle tracking. This framework simultaneously resolves interstitial fluid dynamics and individual nanoparticle (NP) trajectories through coupled solutions of continuum-scale transport and discrete particle motion. Modified collision operators implement multiphysics couplings, incorporating thermomagnetic effects, electrostatic interactions, and short-range surface forces. This study involved comprehensive analyses to elucidate the influence of microenvironmental factors, NP characteristics, and other pivotal parameters on the diffusion and distribution of NPs within tissues, which can contribute to the design of drug-loaded NPs for treatment.

## 2. Modeling and Methods

**Figure 1:**
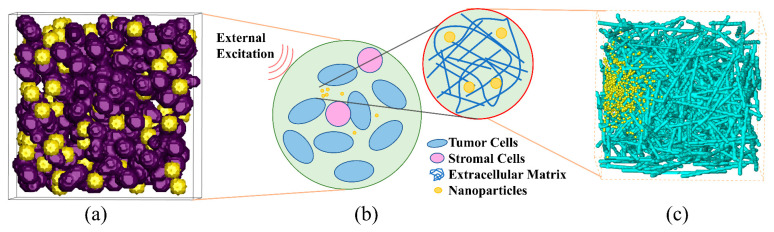
Model of NP transport in porous treated tissues: (**a**) schematic diagram of NPs in porous models of (**b**) tumor tissue and (**c**) ECM.

In this study, we constructed three-dimensional heterogeneous models of tumor tissue (Figure 1a) and the ECM within it (Figure 1c) using the quartet structure generation set algorithm [32]. A schematic diagram depicting the diffusion of NPs in tumor tissues under the influence of an external field is shown in Figure 1b. As depicted in Figure 1a, the tumor cells were ellipsoidal in shape, while the stromal cells were spherical, with a diameter of 10 μm. In the simulation, the ratio of tumor cells to stromal cells was set to 4:1. The computational domain, measuring 100 μm × 100 μm × 100 μm, was discretized into a 100 × 100 × 100 mesh. In the ECM model presented in Figure 1c, collagen fibers are modeled with a diameter of 100 nm and a length of 70 μm. These fibers are randomly distributed within a computational domain measuring 5 μm × 5 μm × 5 μm, which is discretized into a grid of 100 × 100 × 100.

A periodic boundary condition was selected at the boundary of the ECM calculation domain. In the tumor tissue calculation domain, a pressure boundary condition was applied when there was an interstitial pressure gradient, and a periodic boundary condition was applied when there was no pressure gradient. The bounce-back boundary condition is used at the interface between the interstitial fluid and the collagen fiber or cell wall.

Initially, 120 NPs, each 50 nm in diameter, were randomly positioned on the entrance surface of the computational domain for both the tumor and ECM models. The initial velocities of the tumor interstitial fluid in both models were set to 0. This study initially simulated the diffusion of particles within a microscale ECM model. Based on these results, the ECM was approximated as a fluid with an “equivalent viscosity” to analyze particle diffusion among tumor cells. The calculation method and validation of the “equivalent viscosity” will be detailed in subsequent sections. It was assumed that the tumor simulation domain was fully filled with this equivalently viscous fluid.

### 2.1. Coupled Double-Distribution Function Model for Flow and Temperature Fields

To simulate the flow of interstitial fluid and heat transfer in tumor tissues on a mesoscopic scale, we established a three-dimensional coupled-double-distribution-function model using the lattice Boltzmann method. The lattice Boltzmann method (LBM) is well-suited for modeling tumor microenvironments due to its ability, at the mesoscopic scale, to explicitly represent complex boundaries (e.g., irregular cellular geometries, ECM fiber networks) and simultaneously incorporate multiphysics couplings (e.g., thermomagnetic fields, electrostatic interactions) [33,34]. We utilized a two-component BGK–lattice Boltzmann equation (LBE) [35] based on Boussinesq’s assumption, which can be expressed as:

(1) $$f_{\alpha }\left(r+e_{\alpha }\delta_{t},t+\delta_{t}\right)-f_{\alpha }\left(r,t\right)=-\frac{1}{\tau_{f}}\left[f_{\alpha }\left(r,t\right)-f_{\alpha }^{eq}\left(r,t\right)\right]+\delta_{t}F_{\alpha }\left(r,t\right)$$

(2) $$g_{\alpha }\left(r+e_{a}\delta_{t},t+\delta_{t}\right)-g_{\alpha }\left(r,t\right)=\frac{1}{\tau_{g}}\left[g_{\alpha }^{eq}\left(r,t\right)-g_{\alpha }\left(r,t\right)\right]$$

where *f_a_* and ***g_a_*** represent the discretized velocity and temperature distribution functions, respectively, *f_a_^eq^* and *g_a_^eq^* represent their corresponding equilibrium distribution functions, and *τ_f_* and *τ_g_* represent their corresponding relaxation time. The force density term *F_a_* represents the disturbance caused by the temperature difference to the tumor interstitial fluid and can be expressed in terms of the external body force *F = β* (*T_0_* − *T*) *ρg* and fluid macroscale velocity ***u***:

(3) $$F_{\alpha }=\left(1-\frac{1}{2\tau_{f}}\right)\omega_{\alpha }\left[\frac{e_{a}-u}{c_{s}^{2}}+\frac{\left(e_{\alpha }\cdot u\right)}{c_{s}^{4}}e_{\alpha }\right]F$$

where *ρ* and *T*_0_ represent the reference density and temperature, respectively; ***e_a_*** represents the discretized velocity; *c_s_* and *ω_a_* represent the lattice sound speed and the weight coefficient, respectively; and *β* represents the coefficient of thermal expansion.

### 2.2. Modeling of NP Movement

The transport of NPs within tumor tissues is influenced by various factors, such as environmental temperature, high interstitial pressure, cell density, NP–cell interactions, and anisotropic structure. To simulate the movement of NPs in the interstitial fluid of treated tissue, a model was developed using the lattice gas automata (LGA) method [36]. The LGA method enables high-resolution tracking of individual NP trajectories in complex fluidic environments by resolving discrete particle dynamics under multiphysics interactions [37,38]. The coupled LBM–LGA model integrates thermo-hydrodynamic equations with discrete particle kinetics, thereby enabling high-fidelity simulations of NPs diffusion in tumor tissues through explicit modeling of interstitial flow, NP–ECM/cell interactions, and anisotropic microenvironmental constraints. In this model, NPs were treated as mass points due to their significantly smaller size compared to the ECM fibers and tumor cells.

When determining the primary forces acting on the NPs for calculating their displacement in the interstitial fluid, gravity and buoyancy forces were neglected because their magnitudes are much smaller than those of drag force *F_dra_* = *6πμd_p_*(*u* − *u_p_*) caused by the interstitial fluid, Brownian force *F_brown_* = 2*k_B_T*/*d_p_*, and magnetic force *F_mag_* [39], which can be expressed as:

(4) $${\vec{F}}_{mag}=V_{p}M_{sat}\left(\frac{\partial B}{\partial x}{\vec{B}}_{x}+\frac{\partial B}{\partial y}{\vec{B}}_{y}+\frac{\partial B}{\partial z}{\vec{B}}_{z}\right)$$

where *V_p_* is the volume of an NP, *M_sat_* is the NP saturation magnetization intensity per unit volume, and B is the external magnetic field strength.

In the vicinity of cell surfaces in tumor tissues, the interaction forces between NPs and different wall surfaces primarily consist of short-range van der Waals forces *F_LW_* = −*Ad_p_*/12*d*^2^, electrostatic forces *F_EL_* [40], and hydrophilic and hydrophobic forces F_AB_, which dominantly influence the attachment of NPs to the surfaces of ECM collagen fibers or cells. These forces can be expressed as follows:

(5) $$F_{EL}=\pi d_{p}\frac{\kappa \varepsilon \varepsilon_{0}\left[2\Psi_{1}\Psi_{2}-\left(\Psi_{1}^{2}+\Psi_{2}^{2}\right)e^{-\kappa D}\right]}{\left(e^{\kappa D}-e^{-\kappa D}\right)}$$

(6) $$F_{AB}=-\pi d_{p}\Delta G^{AB}\exp\left[\left(d_{0}-d\right)/\lambda \right]$$

(7) $$\Delta G^{AB}=2\left[\sqrt{\gamma_{w}^{+}}\left(\sqrt{\gamma_{1}^{-}}+\sqrt{\gamma_{2}^{-}}-\sqrt{\gamma_{w}^{-}}\right)+\sqrt{\gamma_{w}^{-}}\left(\sqrt{\gamma_{1}^{+}}+\sqrt{\gamma_{2}^{+}}-\sqrt{\gamma_{w}^{+}}\right)+\sqrt{\gamma_{1}^{+}\gamma_{2}^{-}}-\sqrt{\gamma_{1}^{-}\gamma_{2}^{+}}\right]$$

where A is Hamaker’s constant, *Ψ_1_* and *Ψ_2_* are the surface potentials of the NPs and the wall, respectively, *ε* is the dielectric constant of the interstitial fluid, *κ* is the Debye length of the interstitial fluid, *d_0_* is the equilibrium distance, *λ* is the decay length, Δ*G^AB^* is the Lewis acid-base interaction energy, and *γ*^+^ and *γ*^−^ are the electron acceptor parameter and the electron-donor parameter of the surface tension of the material, respectively, which can be measured experimentally.

Thus, the velocity displacement of the NPs can be calculated by *a_p_* = (*u* − *u_p_*)/*τ_p_* + Σ*a*, where *a_p_* and *u_p_* are the acceleration and velocity of the NPs, respectively; (*u* − *u_p_*)/*τ_p_* is the acceleration due to fluid traction; *u* is the local fluid velocity, as described above, computed using LBM; *τ_p_* is the relaxation time of the NP, which can be calculated by *τ_p_* = *ρ_p_d_p_*^2^/18*υ*; and Σ*a* is the sum of the accelerations produced by forces other than fluid drag.

By integrating the equation *a_p_* = d*u_p_*/d*t*, the velocity of the particle can be obtained by Equation (8), and the displacement of the particle can be determined by Equation (9).

(8) $$u_{p}^{*}=u_{p}\exp\left(1-\frac{\Delta t}{\tau_{p}}\right)+\left(u+\tau_{p}\sum a\right)\left[1-\exp\left(-\frac{\Delta t}{\tau_{p}}\right)\right]$$

(9) $$\Delta x_{p}=u\Delta t+\left(u_{p}-u\right)\left[1-\exp\left(-\frac{\Delta t}{\tau_{p}}\right)\right]\tau_{p}+\left\{\Delta t+\tau_{p}\left[1-\exp\left(-\frac{\Delta t}{\tau_{p}}\right)\right]\right\}\tau_{p}\sum a$$

Subsequently, the probability (P_i_) of NP migration to neighboring lattice points and the coordinates of an NP after one time step can be calculated using the following equation:

(10) $$P_{i}=\max\left(0,\left(u_{p}\cdot e_{i}\right)\frac{\Delta t}{\Delta x}\right)=\max\left(0,\frac{\Delta x_{p}}{\Delta x}\cdot e_{i}\right)$$

(11) $$x_{p}^{*}=x_{p}+\sum_{i=1}^{6}\lambda_{i}ce_{i}\Delta t$$

Particle positions were obtained via simulation to compute the mean squared displacement (MSD) and derive the diffusion coefficient. For each condition, 50 independent simulations were performed, and the ensemble diffusion coefficient was calculated by averaging the values from these replicates. The MSD curve presented corresponds to the replicate with a diffusion coefficient closest to the ensemble average. The simulations in this study were conducted using Microsoft Visual Studio 2022, with custom code developed in the C++ programming language.

### 2.3. Model Validation

**Figure 2:**
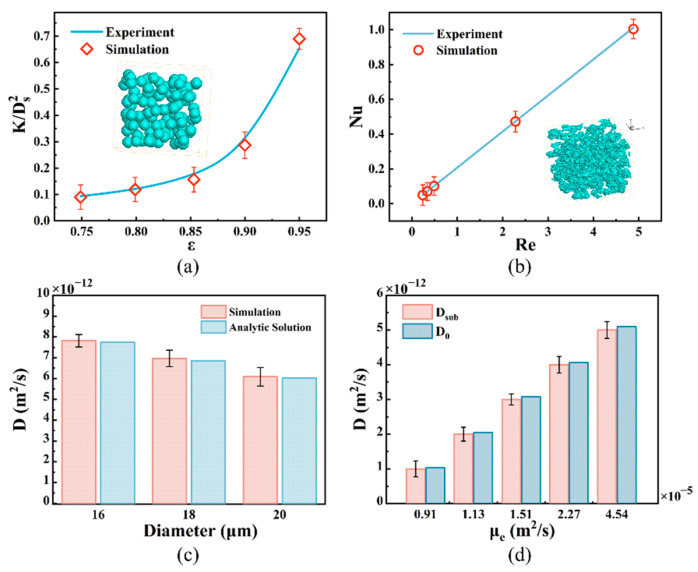
Model validation. All values are shown as mean ± SD. (**a**) Comparison between the relative permeabilities (K/D2) obtained in the experiment [41] and the LB simulation, with the inset figure showing the model used for simulation; (**b**) comparison between the Nusselt numbers under the same Reynolds number (Re) in the experiment [42] and the LB simulation, with the inset figure showing the model used for simulation; (**c**) comparison of diffusion coefficients for regularly arranged spheres with the same diameter between analytical solutions and the LB–LGA simulation results; (**d**) comparison of the subdiffusion coefficient with the free diffusion coefficient corresponding to “equivalent viscosity”.

To validate the accuracy of the established mathematical models used for simulating NP diffusion and heat transfer in porous media models of the ECM and tumor tissues at the mesoscopic scale, we conducted simulations. We simulated the flow of air in a porous spherical skeleton medium and compared it with an empirical equation [41], as depicted in Figure 2a. Additionally, we simulated convective heat transfer within a porous medium and compared it with experimental data [42], as illustrated in Figure 2b. It is evident from the figures that the simulated data align well with the experimental data, thus indicating the reliability of the established model based on the LBM–LGA method. Furthermore, we validated the accuracy of the model by simulating NP diffusion within a porous medium containing regularly arranged spheres of identical diameter, as shown in Figure 2c. The results revealed that the maximum relative error of the diffusion coefficient between the numerical simulation data and theoretically calculated results was 1.45% for media with different ball diameters, thus demonstrating the high accuracy and reliability of the constructed model. The simulation results also showed that the diffusion coefficients of the NPs in the x, y, and z directions in the constructed ECM model were 5.45 × 10^−13^ m^2^/s, 2.40 × 10^−13^ m^2^/s, and 3.61 × 10^−13^ m^2^/s, respectively, and the diffusion coefficients in the x, y, and z directions in the constructed tumor model were 1.40 × 10^−13^ m^2^/s, 1.21 × 10^−13^ m^2^/s, and 1.60 × 10^−13^ m^2^/s, respectively, indicating that there were clear differences in the diffusion coefficient in different directions due to the anisotropy of the ECM and porous media in the tumor tissue models.

In addition, since tumor tissue includes tumor cells and a cellular interstitial space composed of ECM and interstitial fluid, as described above, we introduce the concept of “equivalent viscosity” (*μ_e_* = *kT*/6*πR_0_ D_sub_*, *m*^2^/*s*) to study particle diffusion among tumor cells. This concept equates the viscosity of the ECM and interstitial fluid with that of tumor cells, enabling a simplified model of nanoparticle diffusion between tumor cells. To verify the accuracy of this simplified model, we compared the diffusion coefficients of NPs transported in fluids of different “equivalent viscosities” with those transported in interstitial fluids and the ECM, as depicted in Figure 2d. The free diffusion coefficients of the NPs in the fluid with “equivalent viscosity” exhibited good agreement with the corresponding diffusion coefficients in the actual situation, with a maximum relative error of 3.0%. This indicates that the concept of “equivalent viscosity” can accurately describe the diffusion characteristics of NPs in interstitial fluids and the ECM.

## 3. Results and Analysis

### 3.1. NP Diffusion in Tumor Tissues with Different Structures

**Figure 3:**
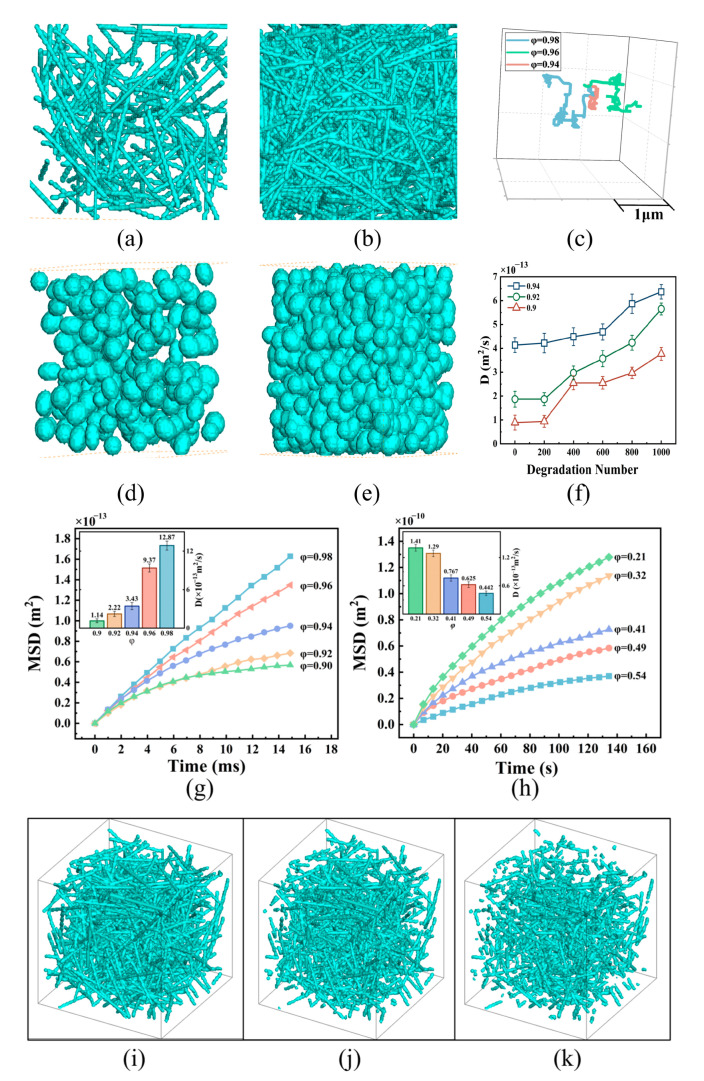
Effect of tissue structure on NP diffusion. ECM structures with porosities of approximately (**a**) 0.98 and (**b**) 0.92; (**c**) motion paths of a single particle in ECMs with different porosities; porous medium models of tumor tissue with cell volume fractions of (**d**) 21% and (**e**) 54%; (**f**) diffusion coefficient profiles of NPs in ECMs with different porosities and degradation points; MSDs and diffusion coefficients of NPs (**g**) in the ECM with different porosities and (**h**) in tumor tissues with different volume fractions; porous medium models of the ECMs: (**i**) undegraded, (**j**) with 600 degradation points on the fibers, and (**k**) with 1000 degradation points. All values are shown as mean ± SD.

To analyze the effect of pore structure on the diffusion characteristics of NPs, we constructed porous media models of ECMs with varying porosities (Figure 3a,b) and tumor tissues with different volume fractions of tumor cells (Figure 3c,d). Using the simulation models described in Section 2, we obtained mean-square displacement (MSD) curves (‹*MSD*›*_ij_* = 2*D_ij_Nt*, where N is the motion dimension correlation coefficient, which is taken as 1, 2, and 3 for one, two, and three dimensions, respectively) for NP diffusion in the ECMs with different porosities and in tumor tissues with different cell volume fractions (Figure 3g,h). The results indicated that higher ECM porosity led to an increase in the MSD of the NPs, and the diffusion coefficient significantly increased with a slight increase in porosity. Specifically, compared to an ECM with a porosity of 0.9, the diffusion coefficients of NPs in ECMs with porosities of 0.92, 0.94, 0.96, and 0.98 were increased 1.9, 3.0, 8.2, and 11.3 times, respectively. This result aligns with the experimental observations reported by Cahn [43]. Cahn et al. employed a decellularized extracellular matrix hydrogel model integrated to systematically evaluate the diffusion kinetics of polyethylene glycol–functionalized nanoparticles under varying ECM concentrations. The model demonstrated that elevated ECM concentrations significantly reduced NP diffusion rates. Additionally, as shown in Figure 3h, the MSD of the NPs decreased with increasing volume fraction of tumor cells in the tumor tissue, resulting in a significant reduction in the diffusion coefficient of the NPs. Specifically, the diffusion coefficient of NPs in tumor tissue with a cell volume fraction of 0.54 was 68.7%, lower than that seen with a cell volume fraction of 0.21. This result is consistent with the findings of Kwak et al. [44]. Kwak et al. employed a tumor-microenvironment-on-chip (T-MOC) model to recapitulate the tumor microenvironment and demonstrated that elevated cell packing density significantly restricted NP transport. This reduction can be attributed to a decrease in the flow area of tumor tissues and a decrease in pore connectivity caused by the decrease in porosity. These factors increase the likelihood of NP margination to the cell walls and subsequently facilitate NP attachment, weakening the diffusion of NPs.

To improve the diffusion of NPs in tumor tissues, a possible solution is to enhance tissue connectivity by breaking and degrading the porous framework using local heating and mechanical loading. A “degradation point” is defined as the site where ECM fibers, measuring 200 nm in length, undergo degradation. Figure 3i–k show ECM geometries with a porosity of 0.94 and varying numbers of “degradation points”. In these models, “degradation points” are randomly distributed across all ECM fibers. The results indicate that increasing the number of degradation points enhances NP diffusion in the ECM (Figure 3f). The diffusion coefficient for the case with 1000 degradation points is 1.54 times greater than that for the case without degradation. This result is consistent with the experimental findings of Raeesi et al. [45], who used near-infrared–activated gold nanorods to degrade the collagen matrix, thereby enhancing NP diffusion.

However, the diffusion coefficient does not significantly increase with further increases in degradation points when the number of pores is less than 200. This may be because a small number of degradation points is insufficient to noticeably enhance the connectivity of the porous structure in biological tissues. Thus, a certain number of degradation points is necessary to improve NP diffusion. However, from a biological perspective, excessive ECM degradation reduces tumor tissue stiffness, facilitating cancer cell migration and metastasis [46]. Furthermore, it may release pro-inflammatory factors that trigger local immune responses or chronic inflammation, potentially compromising therapeutic efficacy or inducing adverse effects [47].

### 3.2. Effects of NP Surface Properties

**Figure 4:**
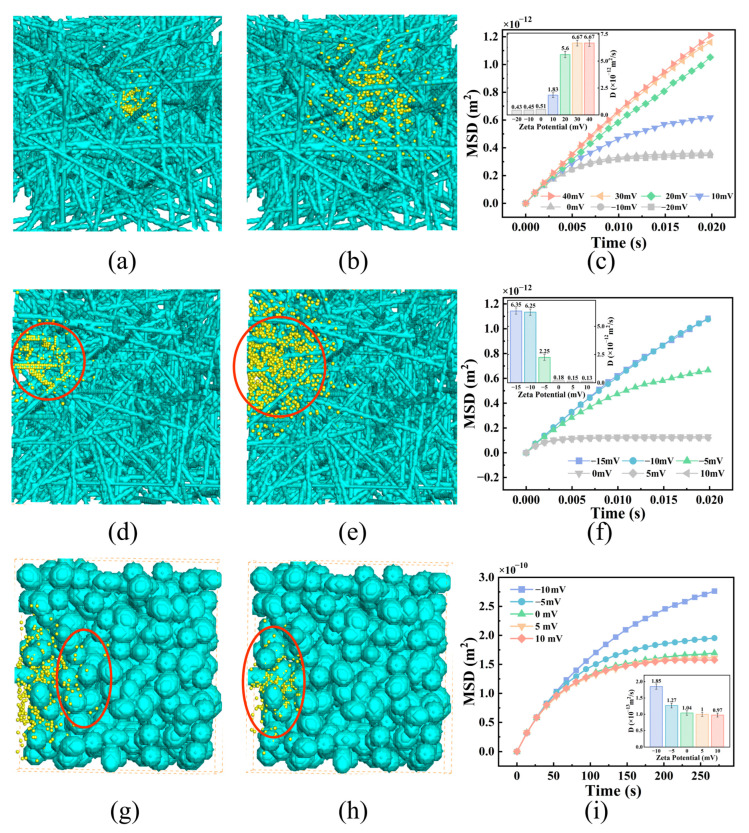
Effect of NP zeta potential on NP diffusion. The red circle highlights the location of the nanoparticles. (**a**,**b**) Diffusion in the ECM with hyaluronic acid fibers for NPs with zeta potentials of 10 mV and −10 mV; (**c**,**f**) MSD curves and diffusion coefficients of NPs with different surface zeta potentials in the ECM with hyaluronic acid fibers and collagen fibers; (**d**,**e**) diffusion of NPs in the ECM with collagen fibers for NPs with zeta potentials of 20 mV and −20 mV; (**g**,**h**) diffusion among tumor cells with zeta potentials of −10 mV and 10 mV; and (**i**) MSD curves and diffusion coefficients of NPs with different surface zeta potentials among tumor cells. All values are shown as mean ± SD.

The zeta potential and surface hydrophilicity of the NP surface may have a significant impact on NP diffusion within tumor tissues. ECM fibers consist of positively charged collagen and negatively charged hyaluronic acid, and their proportions vary depending on the stage of tumor growth. In this study, we conducted simulations to investigate the influence of NP zeta potential and surface hydrophilicity on NP diffusion within an ECM containing either collagen fibers or hyaluronic acid fibers. Our findings revealed that, compared with NPs with a low negative surface charge, NPs with a high negative surface charge demonstrated greater diffusion coefficients in the ECM with hyaluronic acid fibers (Figure 4c). Moreover, the diffusion coefficients of the NPs with surface zeta potentials of −15 mV, −10 mV, and −5 mV in the ECM were 35.3, 34.7, and 12.5 times greater than those of NPs with a surface zeta potential of 0 mV, respectively. As the negative surface charge of the NPs increased, the repulsive force between the NPs and the fiber surface became stronger, surpassing the attractive force caused by van der Waals interactions between the fibers and the NPs. This prevented the NPs from adhering to the fiber wall and likely explains why NPs with surface zeta potentials of −15 mV and −10 mV exhibited similar diffusion coefficients. Similarly, as shown in Figure 4f, the diffusion of positively charged NPs within ECMs containing positively charged collagen increased with increasing NP zeta potential. The diffusion coefficients of the NPs with surface zeta potentials of 10 mV and 20 mV in the ECM were 3.6 and 11.0 times greater than those of the NPs with a surface zeta potential of 0 mV, respectively.

To enhance NP diffusion in tumor tissues, the surface properties of NPs can be altered by modification of the NP surface via a localized enzymatic response to a specific substance. In our simulation, we assumed that the NPs were electrically neutral in the interstitial fluid. However, they became negatively or positively charged upon reaching the vicinity of tumor cell membranes, which typically carry a negative charge [48]. As demonstrated in Figure 4g–i, NPs with negative zeta potentials exhibited superior diffusion characteristics in tumor tissues compared to NPs with positive zeta potentials or a neutral surface. The diffusion coefficients of the NPs with zeta potentials of −10 mV and −5 mV were 1.8 and 1.2 times greater, respectively, than those of the NPs with a zeta potential of 0 mV. These findings indicate that the influence of NP zeta potential on diffusion in tumor tissue mirrors that in the ECM. The adhesion of NPs to tumor cell membranes was strengthened by the attractive force between opposite charges, thus impeding NP diffusion. Conversely, the repulsive force between similar charges could weaken this adhesion. These results are consistent with the experimental findings of Wang et al. [49]. Wang et al. constructed an in vitro tumor vascular barrier model using human microvascular endothelial cells (HMVECs) and demonstrated that negatively charged Au NPs exhibited enhanced transendothelial diffusion by localizing at cell–cell junctions, whereas positively charged NPs were retained on the endothelial surface and rapidly internalized, thereby limiting their diffusion. However, when the repulsive force surpassed the van der Waals force by increasing the NP zeta potential to a certain value, further increases in the NP zeta potential no longer enhanced the diffusion ability of the NPs.

**Figure 5:**
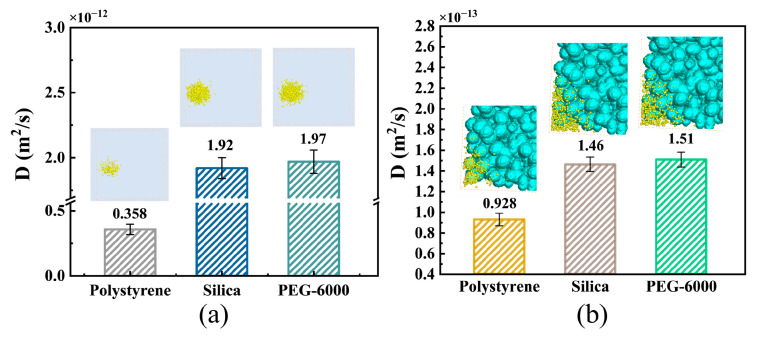
Effect of NP surface hydrophilicity on NP diffusion (**a**) in the ECM and (**b**) among the tumor cells. All values are shown as mean ± SD.

To explore the effect of hydrophilic or hydrophobic interactions between NPs and porous surfaces, it is important to understand how these interactions affect the diffusion of NPs. In this study, we investigated the effect of NP surface hydrophilicity/hydrophobicity on NP diffusion in tumor tissues. Specifically, we simulated the diffusion behaviors of hydrophobic polystyrene, hydrophilic PEG-6000, and hydrophilic silica NPs in ECM with hydrophilic hyaluronic acid fibers, as well as among tumor cells with membranes containing hydrophilic dextran. Figure 5a clearly illustrates that the diffusion of NPs with hydrophilic surfaces in the ECM was greater than that of NPs with hydrophobic surfaces. In fact, the diffusion coefficient of the hydrophilic silica NPs was approximately 5.36 times greater than that of the hydrophobic polystyrene NPs. Similarly, as shown in Figure 5b, we observed that the diffusion of hydrophilic silica NPs among tumor cells was greater than that of hydrophobic polystyrene NPs. The diffusion coefficient of hydrophilic silica NPs in tumor cells was 1.6 times greater than that of hydrophobic polystyrene NPs. Furthermore, we calculated the short-range interaction forces between different NPs and hyaluronic acid using molecular dynamics methods. The interaction forces between polystyrene, silica, and PEG-6000 NPs were determined to be −6.46 × 10^−10^ N, 1.12 × 10^−10^ N, and 1.12 × 10^−9^ N, respectively. Similarly, we calculated the interaction forces between different NPs and dextran, which were −4.96 × 10^−10^ N, 6.40 × 10^−10^ N, and 1.08 × 10^−9^ N for polystyrene, silica, and PEG-6000 NPs, respectively. Our findings indicate that hydrophobic polystyrene NPs can exhibit mutual attraction with hyaluronic acid fibers in the ECM or dextran on cell membranes, leading to enhanced NP adhesion to the ECM fiber wall or tumor cell membrane. Consequently, this adhesion weakens NP diffusion in tumor tissues. Moreover, the adhesion of certain NPs may cause a decrease in the connectivity of the ECM or porous tumor structure, thereby inhibiting the diffusion of other NPs. In contrast, hydrophilic polystyrene silica or PEG-6000 NPs exhibit mutual repulsion with hyaluronic acid fibers in the ECM or dextran on cell membranes. This repulsion reduces the number of adhered NPs on the ECM fiber surface and cell membrane. As a result, this facilitates deeper diffusion of NPs into the tumor tissues. This result aligns with experimental findings reported by Cahn et al. [43], who showed that PEGylation significantly improved nanoparticle diffusion within ECM hydrogels.

### 3.3. Changes in NP Diffusion Caused by External Fields

**Figure 6:**
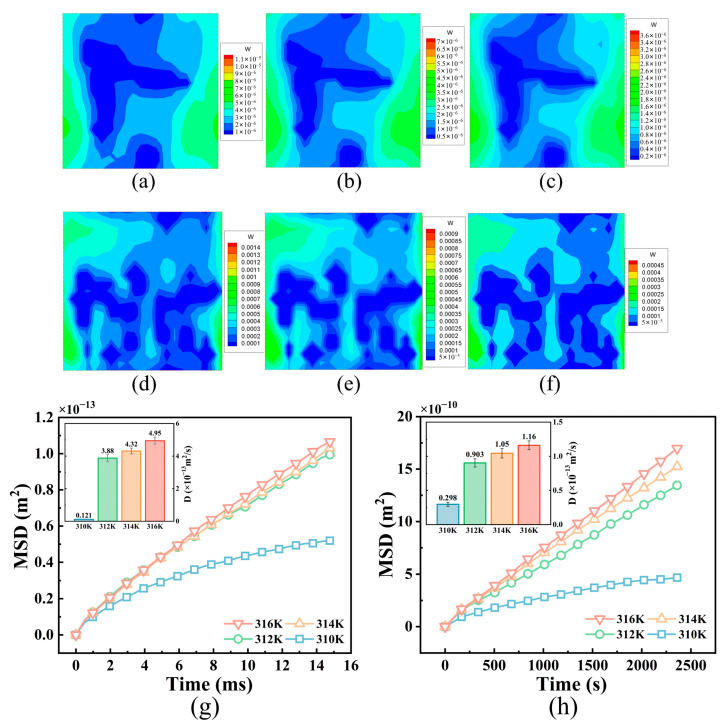
Effect of external fields on NP diffusion. Velocity distributions of interstitial fluid in the z-direction at NP temperatures of 316 K, 314 K, and 312 K: (**a**–**c**) in the ECM and (**d**–**f**) among tumor cells; MSD curves and diffusion coefficients of NPs at different temperatures: (**g**) in the ECM and (**h**) among tumor cells. All values are shown as mean ± SD.

Some investigations have demonstrated that the diffusion of NPs within tumor tissue can be facilitated by the application of an infrared field, an alternating magnetic field, or other external fields that induce heat generation [14]. The objective of this study was to examine the diffusion characteristics of NPs at various NP temperatures in both the ECM and among tumor cells. Figure 6a–f display the velocity distributions for the fluid in the ECM in the z-direction at NP temperatures of 316 K, 314 K, and 312 K, respectively, as well as for the interstitial fluid between tumor cells. The diffusion coefficients of the NPs increased both in the ECM and among the tumor cells with increasing NP temperature (Figure 6g,h), with the maximum increase in the diffusion coefficient exceeding threefold. Previous studies have suggested that, under normal temperature conditions, there is minimal interstitial fluid flow throughout the tumor due to functional impairment of lymphatic vessels [14]. However, our simulation revealed that NP heat generation facilitated local fluid flow within the tumor tissue. Higher NP temperatures resulted in stronger natural convection of the interstitial fluid due to increased drag force acting on the NPs, thereby promoting transport of the NPs in the direction of flow. This result is consistent with the experimental findings of Zhang et al. [50], who showed that magnetic heating of nanoparticles under an alternating magnetic field significantly enhances their diffusion efficiency in tumor tissues.

**Figure 7:**
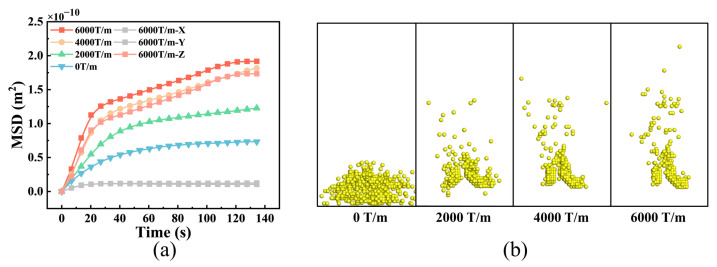
Effect of external fields on NP diffusion. (**a**) MSD curves of NPs in tumor tissues under different magnetic field intensity gradients; (**b**) NP distributions in the z-direction in the tumor tissue under magnetic field intensity gradients of 0 T/m, 2000 T/m, 4000 T/m, and 6000 T/m. The background color visually represents the distribution of magnetic field strength.

In addition, efforts have been made to improve the diffusion of magnetic NPs in tumor tissues by applying a static gradient magnetic field [51]. To investigate the diffusion mechanism and characteristics of magnetic NPs in tumor tissues, we developed an NP movement model under the influence of magnetic fields (Section 2.2) and conducted numerical simulations. Figure 7a shows curves representing the total MSD of the NPs under different magnetic field gradients, as well as the MSDs in three orthogonal directions under a magnetic field of 6000 T/m. The results demonstrate that the NPs initially diffused freely in the tumor tissue, but their diffusion was subsequently hindered due to an increase in the number of NPs adhering to the cell wall. This phenomenon was caused by the interaction between the NPs and tumor cell membranes. The changes in the MSD pattern depicted in Figure 7a reveal that the MSD of the NPs initially increases linearly and then gradually decreases until it stabilizes. An increase in the magnetic field gradient results in an increase in the curvature and stabilization of the MSD curve of the NPs. By comparing the MSD values of the magnetic NPs in the x-, y- and z-directions at a magnetic field intensity gradient of 6000 T/m, we observed that the MSD along the direction of the field intensity gradient (z-direction) was significantly larger than that in the other two directions. This suggests that the dominant force during NP diffusion in tumor tissues, when a static gradient magnetic field is present, is the magnetic force acting on the NPs. Furthermore, it was evident that there was a similar trend in the changes between the total MSD of the magnetic NPs and the MSD in the z-direction at a field intensity gradient of 6000 T/m. This finding indicates that the enhancement of NP diffusion in tumor tissues mostly results from the magnetic field force acting in the z-direction.

Figure 7b illustrates the spatial distribution of magnetic NPs in tumor tissue under various field intensity gradients. The images were modified to remove tumor cells, ensuring clear observation. The results revealed that the magnetic NPs were predominantly localized near their initial positions, with no significant variation in diffusion among the three orthogonal directions when the field intensity gradient was zero.

With increasing field intensity, the diffusion depth of the magnetic NPs in the Z direction increased, while the diffusion in the x- and y-directions decreased. Furthermore, adhesion of the NPs to the cell membrane gradually weakened, except for those NPs that adhered to the surface opposite the tumor cells in the z-direction. An increase in the magnetic field intensity caused an increase in the magnetic field force acting on the NPs, resulting in the detachment of more NPs from the cell membrane. This detachment facilitated the continued transport of NPs in the z-direction. Therefore, by adjusting the direction and intensity of the magnetic field, transport of NPs in tumor tissues can be improved. It should be noted that the application of a static gradient magnetic field in a single direction to tumor tissue could expand the distribution range of magnetic NPs in that direction. However, it did not enhance uniform distribution of magnetic NPs throughout the entire tumor tissue.

## 4. Conclusions

We developed three-dimensional anisotropic geometric models of ECM and tumor tissues. Mathematical models based on LBM–LGA were then established to describe the movement of NPs within these porous tissues under the effects of temperature and magnetic fields. Our findings indicate that the forces acting on NPs during the diffusion process mainly consist of flow drag forces, van der Waals forces, electrostatic forces, hydrophilic and hydrophobic interactions, and external fields. Through a series of simulations, we discovered that increasing the connectivity of ECM pores, achieved by degrading ECM fibers through heating or pulsed high-intensity focused ultrasonography, significantly enhances the diffusion of NPs. Another major factor affecting NP diffusion is porosity. The presence of opposite charges creates electrostatic repulsion, reducing NP adherence to the wall surface of ECM fibers or cells and resulting in deeper NP diffusion. Regulating the surface zeta potential of NPs also improves their diffusion in tumor tissues. However, once there is enough electrostatic repulsion, further enhancement of NP diffusion becomes limited due to reduced adherence between the NPs and cell walls. Additionally, the natural convection of interstitial fluid in tumor tissues is enhanced by particle-induced heating, leading to improved NP diffusion with the flow of interstitial fluid. By applying a gradient static magnetic field, the penetration depth of NPs along the direction of the magnetic field gradient can be significantly increased. In summary, our approach provides insight into the mechanism of NP diffusion and transport characteristics in tumor tissues. These findings can guide the design of nanodrug carriers and the application of external fields.

There are still some aspects to be addressed in our future work. One is to focus on building a more realistic tumor model and more accurately reproducing the tumor microenvironment, including vascular structure and cell–cell interactions, to more realistically reflect the behavior of NPs. To achieve this goal, we will integrate advanced imaging data and computational modeling techniques to simulate the complex tumor vascular network and its heterogeneity. Specifically, we plan to capture the spatial distribution of blood vessels through high-resolution imaging and employ both agent-based and continuum modeling to simulate dynamic cell–cell interactions within the tumor microenvironment, thereby more precisely predicting NP behavior under clinical conditions. Another aspect to consider is the biological effects of different treatments. This can help us construct a more comprehensive NP prediction model, which can better reflect the actual clinical situation, thereby improving the development and application of nanomedicines.

## Data Availability

The raw data supporting the conclusions of this article will be made available by the authors on request.

## Abbreviations

The following abbreviations are used in this manuscript:

NP	nanoparticle
ECM	extracellular matrix
LBM	lattice Boltzmann method
LGA	lattice gas automata
MSD	mean-square displacement
